# Twenty-year changes of adolescent mental health and substance use: a Finnish population-based time-trend study

**DOI:** 10.1007/s00787-024-02512-9

**Published:** 2024-07-10

**Authors:** Kaisa Mishina, Emmi Heinonen, Lotta Lempinen, Andre Sourander

**Affiliations:** 1 Research Centre for Child Psychiatry, University of Turku, Turku, 20014 Finland; 2 Invest Research Flagship, University of Turku, Turku, Finland; 3 Department of Child Psychiatry, Turku University Hospital, Turku, Finland

**Keywords:** Adolescent, Alcohol, Mental health, SDQ, Smoking, Time-trend analysis

## Abstract

This time-trend study assesses changes in mental health and substance use among Finnish adolescents from 1998 to 2018. Representative samples of adolescents (*N* = 6,600) aged 13–16 years participated in school-based, almost identical cross-sectional studies in 1998 (*n* = 1,446), 2008 (*n* = 2,009), 2014 (*n* = 1,800) and 2018 (*n* = 1,345), respectively. The Strengths and Difficulties Questionnaire was used to assess mental health. When comparing mental health in the clinical range between 1998 and 2018, the main finding was the significant increase of emotional symptoms among females. The percentage of females in the clinical range increased from 17.5 to 30.1% during the twenty-year period. When psychopathology measures were analyzed as continuous variables, the finding of increased emotional problems was confirmed. The study clearly illustrates a linear trend, with a consistent increase in emotional problems among females and decrease in substance use among both genders. An alarming finding of steady increase of self-reported emotional problems indicates the importance of early detection and evidence-based interventions for adolescent with anxiety and depression to prevent adversities associated with these disorders.

**Supplementary Information:**

The online version contains supplementary material available at 10.1007/s00787-024-02512-9.

## Introduction

There has been considerable interest in understanding how changes in society, education systems, youth culture, leisure time and family life affect the mental health of adolescents. It is estimated that nearly one in five young people in Europe have a mental disorder [1]. There is also evidence of an increase in clinical diagnoses [2] and treatment rates of attention-deficit/hyperactivity disorder (ADHD) [3]. Studies have shown increases in the use of mental health services by adolescents [3, 4] and that adolescents are increasingly being prescribed antidepressants [5], antipsychotics [6], and ADHD medication [7]. Possible explanations for these changes include a decrease in stigma around seeking help for mental health problems [8], improved clinical recognition [9], changes in attitudes towards psychiatric and psychopharmacological treatment practices [10], a broadening of diagnostic classifications of adolescent mental health problems and increased recognition by professionals [11]. However, it is also possible that there have been actual changes in the prevalence of adolescent mental health problems.

Time-trend studies include data that have been collected at different time points among similar-aged population in the same geographical area, using the same sampling design and measures. These studies can provide information on whether adolescent psychosocial problems have changed over time [12]. This information is crucial as the growing number of psychiatric clinical diagnoses and smoking and alcohol habits among adolescents are a public health concern [13]. It is important to understand the trends and state of adolescents’ mental health and well-being so that we can target population-based health priorities and improve early interventions and mental health and substance use services for adolescents.

A groundbreaking book published by Rutter and Smith in 1995 [14] assessed changes in children’s and adolescents’ psychiatric problems and concluded that there had been increases in depression, conduct problems, substance use and suicide among adolescents. In 2015 Collishaw [12] published an extensive systematic review on the changes in psychiatric problems among children and adolescents. This review [12] supported real increases in the prevalence of affective symptoms. The review found increases in antisocial behavior among young people from the 1970s to the early 1990s in high income countries, but later these trends had decreased in many countries [12]. Annual review reported that internalizing symptoms and psychological distress have considerably increased among adolescents in many countries, especially among females [15]. Based on the Health Behaviour in School-aged Children study, in most of the Nordic countries, girls have worse mental well-being than boys [16]. Specifically, the School Health Promotion study on Finnish children and adolescents reported significant increases in anxiety and depressive symptoms among females and males, but rates and increases were especially high among females [17]. In addition, alcohol use and smoking among adolescents has decreased in many European countries since 2000 [18, 19].

Many previous time-trend studies have either focused only on two assessment points, the interval between the cross-sectional studies has been rather short, or they have faced other methodological challenges [12]. Optimally, studies assessing secular changes in adolescent mental health in a population should use the same study designs, methods, and recruitment areas at different timepoints. These should also cover the same age ranges and have sufficient intervals between timepoints [18].

This study explored trends in self-reported mental health problems and substance use among adolescents over a 20-year period, from 1998 to 2018. The study involves four population-based, cross-sectional samples, taken at four different time points, the design and methods of which were almost identical. The information includes validated measure of psychopathology. The samples were taken from northern and southern Finland, which makes the findings more generalizable. Based on previous research [12], we expected to see an increase in internalizing problems and, particularly, a decrease in smoking, due to large nationwide anti-smoking campaigns in Finland.

## Methods

### Participants and study procedure

This time-trend study included data from four cross-sectional surveys conducted in two Finnish cities, Rovaniemi and Salo, in 1998, 2008, 2014 and 2018. Rovaniemi is located in the northern part and Salo in the southern part of Finland. The characteristics of the populations living in Rovaniemi and Salo, with regard to population structure, mean age, gender distribution, educational structure, income distribution, ethnic background, and family composition, were comparable to the general population in Finland [20] (Supplement S1).

The Ethical Committee at the Hospital District of Turku University Hospital (1998, 2008) and The Ethics Committee of the University of Turku (2014, 2018) approved the study, and permission was obtained from school authorities. Participation was voluntary for adolescents, and their anonymity was ensured. Parental consent was requested through informing them beforehand about the study, and they had the possibility to not let their adolescent participate in the study.

The data were collected from all secondary schools in these two cities, excluding schools and classes intended for children with special needs. Due to changes to municipality structures in Finland, Rovaniemi and Salo have merged with other municipalities since the start of this study. Only the municipalities that provided data from each of the four measurement points were included in the analysis.

The study participants were adolescents in the 7th or 9th grade in Finnish secondary school, comprising adolescents aged 13–16 years (few students were as old as 17). To ensure confidentiality in the study, the adolescents filled out the questionnaires anonymously during a school lesson and returned them to the teacher in sealed envelopes. These were then placed in another envelope and returned to the research group. The teachers were informed about the confidential nature of the study, and they took the necessary precautions during the school lesson.

**Fig. 1:**
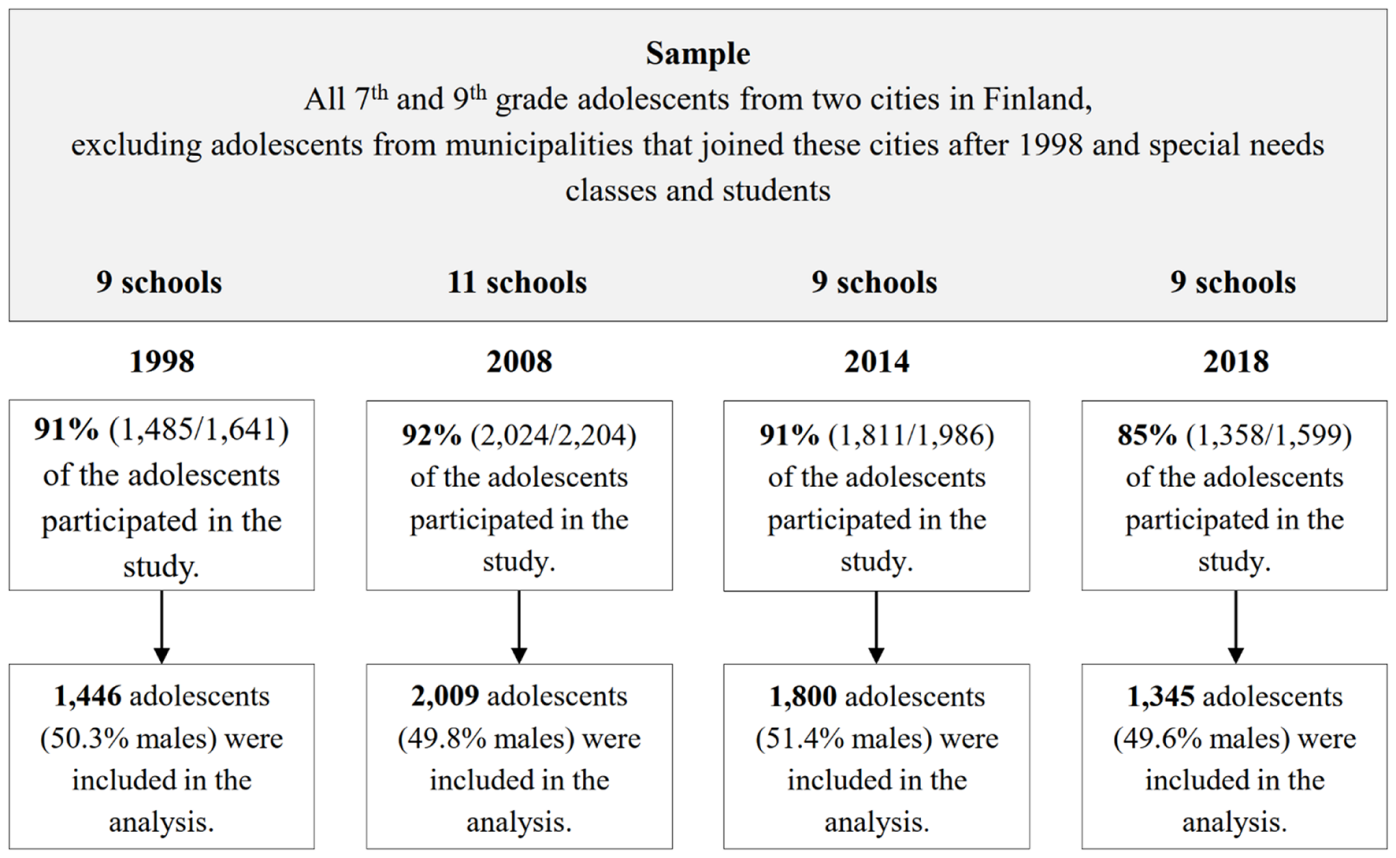
Flow chart

Teachers were instructed to ask any absent students to fill out the questionnaire at a later date in the same conditions (at school) as the respondents on the day of the survey. Despite reminders, there were non-respondents who had been absent from school that day and never completed the survey. There were also students and classes who did not want to participate. In 1998, 156 adolescents did not participate; 180 in 2008; 175 in 2014; and 241 in 2018. This resulted in a total of 6,678 returned questionnaires. Of those, 78 were excluded due to incomplete or inappropriate answering: 39 in 1998; 15 in 2008; 11 in 2014; and 13 in 2018. This left us a total of 6,600 adolescents in the final sample (Fig. 1).

### Measures

*Demographic details* included age, school grade, city, gender, family structure and ethnic background. Family structure comprised six categories: living with two biological parents, one biological parent, remarried parents, foster parents, adoptive parents, or others. When family structure was used as a covariate in the adjusted analyses, it was divided into two categories: two biological parents and other. Ethnic background included information about the adolescents (I was born in Finland; My native language is Finnish) and their parents (My biological mother/father was born in Finland; His/Her native language is Finnish) in terms of their place of birth and native language.

*Mental health* was assessed with the Strengths and Difficulties Questionnaire (SDQ), assessing positive and negative behaviors, emotions and relationships. In this study, a double-translated Finnish version of the questionnaire was used [21]. There are 25 items divided between five scales: emotional symptoms, conduct problems, hyperactivity, problems with peers, and prosocial behavior [22, 23]. The possible scores for each scale of five items could range from 0 to 10. Using a three-point scale (0 = not true, 1 = somewhat true, and 2 = certainly true), respondents were asked to indicate how things had been for them over the last six months. Five items were worded positively and scored in the opposite direction. Four of the scales, not including the prosocial scale, were added together to provide a total difficulties score of 0–40, with higher scores indicating more symptoms. Of the 25 items, 10 items reflected strengths, 14 items reflect difficulties, and one item from the peer problems subscale, “I get on better with adults than with people my own age”, was neutral but scored as a difficulty item. To define any groups with the most severe problems, the cut-off points of the 90th percentile for the SDQ total scores and sub-scales were used, based on the previous European studies [19, 21–27] and on a Finnish normative sample from 1998 [24]. The correlations between the sub-scales were moderate, and Cronbach’s alphas varied between 0.55 and 0.70 (Supplement S2).

*Substance use* was assessed by asking “How often do you use alcohol [e.g., beer]?” and “How often do you use enough alcohol to get drunk?”, with the responses of “not at all”, “once a month or more often”, and “once a week or more often”. Smoking was assessed by asking “How often do you smoke cigarettes or use other nicotine products?”, with the responses being “not at all”, “not often”, “every week”, and “every day”. In the analyses, the responses “every week” and “every day” were combined as “at least once a week”.

### Statistical method

In order to take variation between schools into account, mixed effects models with school-wise random intercepts were used to examine changes in outcomes between study years. SDQ scales were examined both as categorical and continuous variables. For total difficulties, emotional symptoms, conduct problems, hyperactivity and peer problems, mixed effects logistic regression was used to estimate the probability of a score equal to or higher than the 90th percentile cut-off point (calculated from the 1998 sample). Because a low prosocial score indicates problems, mixed effects logistic regression was used to estimate the probability of a prosocial score equal to or lower than the 10th percentile cut-off point. The means of continuous SDQ scores were compared with mixed effects linear regression. Changes in substance use were examined using mixed effects multinomial logistic regression with the option “not at all” as the reference category. Equations for the models in Supplement S3.

Association of interaction of year and sex with categorical SDQ scales and substance use was tested for with mixed logistic regression (binary or multinomial). Because differences in the effect of year were found for some outcomes, all further analyses were conducted separately for males and females. Odds ratios and differences of means were calculated for 2008 vs. 1998, 2014 vs. 2008, 2018 vs. 2014 and 2018 vs. 1998 with Bonferroni correction to control the overall probability of false positive results. This meant that a p-value lower than 0.05/4 = 0.013 was considered significant for one pairwise comparison, and estimates were calculated with a 98.75% confidence interval. Type 3 tests were performed to estimate the overall significance of the year as an explanatory variable. An overall p-value of less than 0.05 was considered significant, meaning that the average outcome was not similar in all years. All the results except background characteristics were adjusted by school grade, family structure and city.

To examine the overall yearly change in outcomes during the 20-year period, all analyses described above were repeated with the study year as a continuous explanatory variable centered on 1998. Odds ratios and differences of means between two consecutive years were estimated with 95% confidence intervals.

The statistical analyses were conducted using SAS 9.4 for Windows.

## Results

The participants of each study (1998, 2008, 2014 and 2018) were similar in age, school grade division and gender (Table 1). Ethnical background was only asked about from 2008 onwards. The number of participants with a biological parent born somewhere other than Finland increased significantly from 2008 to 2018 (mother OR 0.7, 97.5% CI 0.51–0.96, father OR 0.7, 97.5% CI 0.52–0.99).

**Table 1:** Background characteristics of the participants in 1998, 2008, 2014 and 2018

Characteristics	1998 (*N* = 1,446)	2008 (*N* = 2,009)	2014 (*N* = 1,800)	2018 (*N* = 1,345)
City *n* (%)				
Rovaniemi	799 (55.3)	1,286 (64.0)	1,067 (59.3)	767 (57.0)
Salo	647 (44.7)	723 (36.0)	733 (40.7)	578 (43.0)
Age (years)				
mean (SD)	14.4 (1.1)	14.4 (1.1)	14.3 (1.1)	14.5 (1.1)
min-max	13–17	13–17	13–17	13–17
**School grade n (%)**				
7th graders	719 (49.7)	1,053 (52.4)	926 (51.4)	666 (49.5)
9th graders	727 (50.3)	956 (47.6)	874 (48.6)	679 (50.5)
**Gender n (%)**				
Females	719 (49.7)	1,008 (50.2)	874 (48.6)	678 (50.4)
Males	727 (50.3)	1,001 (49.8)	926 (51.4)	667 (49.6)
**Family structure n (%)**				
Family with two biological parents Single-parent or reconstituted family Other family	1,008 (70.4) 398 (27.8) 26 (1.8)	1,323 (66.1) 646 (32.3) 33 (1.6)	1,256 (70.6) 489 (27.5) 34 (1.9)	726 (70.6) 281 (27.3) 22 (2.1)
**Ethnical background n (%)**				
Born in Finland Finnish as native language Biological mother born in Finland Biological father born in Finland	- - - -	1,932 (96.7) 1,934 (97.1) 1,887 (94.6) 1,888 (94.8)	1,723 (96.2) 1,737 (97.0) 1,681 (93.9) 1,677 (94.1)	1,275 (95.3) 1,278 (95.7) 1,224 (91.5) 1,225 (91.9)

### Changes in mental health

Changes in mental health were assessed with subscales of SDQ assessing both, overall trend and pairwise year comparisons. Both the linear results and logistic models were adjusted for school grade, family structure and city. After adjustment, the results remained unchanged when comparing the years 1998 and 2018. The results from all the years are presented in the Supplement S4 and S5 and in figure, but the text only describes the results between the first and last measures (1998 and 2018).

**Fig. 2:**
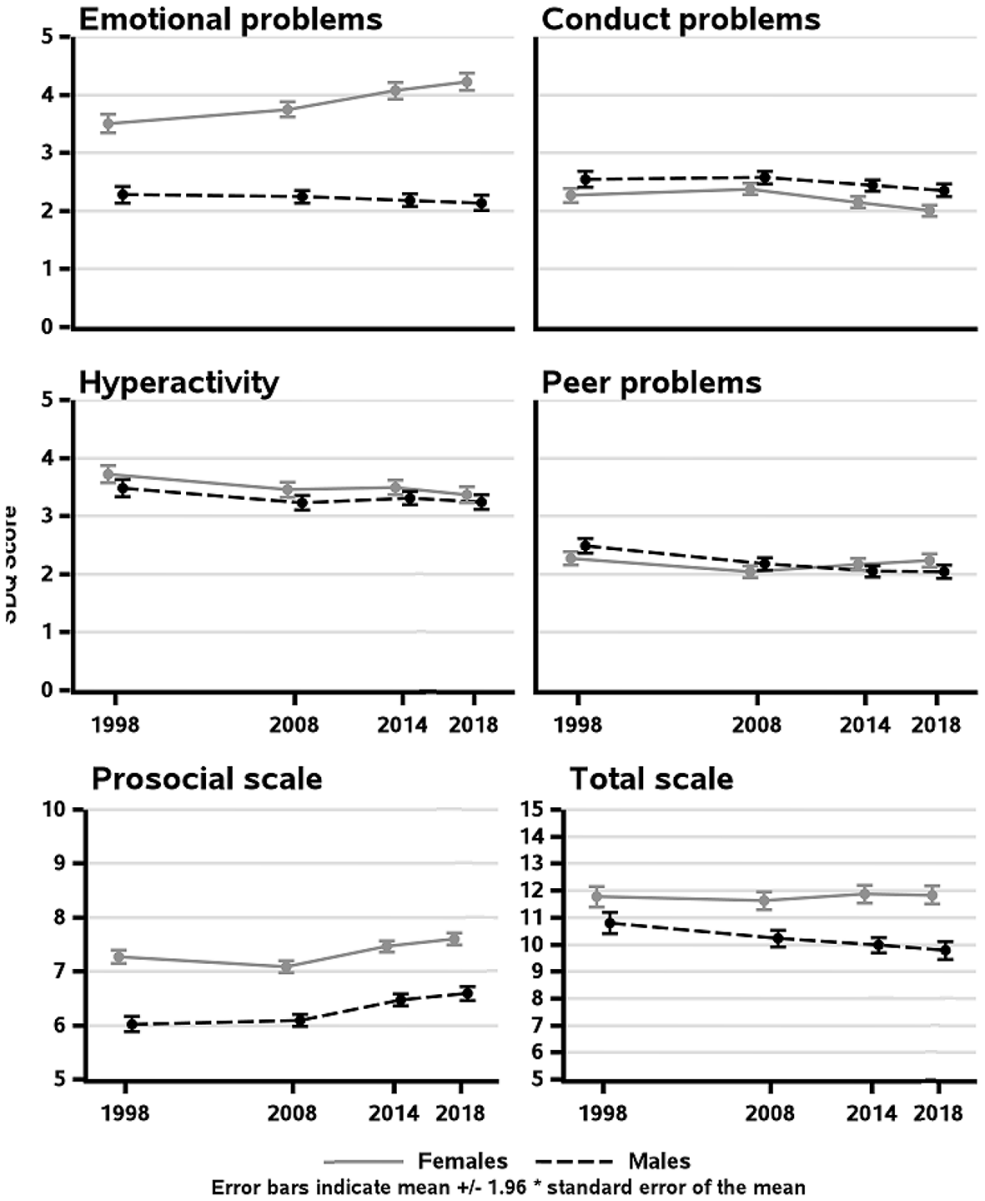
Mean scores of the SDQ in 1998, 2008, 2014 and 2018

Changes in the mean scores of mental health problems are presented in Fig. 2, and mean scores of mental health problems are presented in Supplement S4. When comparing the years 1998 and 2018 among females, mean scores in emotional symptoms increased from 3.5 (SD = 2.2) in 1998 to 4.3 (SD = 2.4) in 2018 (*p* < 0.001). Similar change was not found among males. Mean scores in prosocial behavior increased among males from 6.0 (SD = 1.9) in 1998 to 6.6 (SD = 2.0) in 2018, (*p* < 0.001) and the mean scores in peer problems decreased from 2.5 (SD = 1.7) in 1998 to 2.0 (SD = 1.8) in 2018 (*p* = 0.002).

In the mean scores of mental health problems assessed using categorical years, an interaction was found between year and gender for total difficulties (interaction sex × year p-value 0.01), emotional problems (interaction sex × year p-value 0.001), prosocial behavior (interaction sex × year p-value 0.08), peer problems (interaction sex × year p-value 0.001). Interactions followed similar statistical significance levels in linear and in categorical year assessments. Total difficulties decreased among males and were stable among females. Emotional problems increased among females, while they remained rather stable among males. Peer problems and prosocial behavior slightly increased and was rather stable among females.

Changes in self-reported mental health problems are presented in Table 2, using the 90th percentile cut-off points (Supplement S6 for subscales 90th percentiles in each year). When comparing 1998 and 2018, there were significantly more females with a clinical range of emotional symptoms in 2018 than in 1998 (OR 2.1, 98.75% CI 1.51–3.05). Correspondingly, continuously increased linear trend was seen when using year as a continuous explanatory variable (OR 1.04, 98.75% CI 1.03–1.05). No other significant changes among the mental health symptoms of females or of males were found when comparing 1998 and 2018.

**Table 2:** Comparison of adjusted self-reported problems based on 90th percentile cut-off points of SDQ and substance use in 1998, 2008, 2014 and 2018

	1998	2008	2014	2018	2018 vs. 1998	Overall year *p*-value^a^		Year as continuous explanatory variable OR (95%CI)	*p*-value
	%	%	%	%	OR (98.75%CI)^a, d^				
**SDQ** ^**c**^ **total score ≥ cut-off**									
Females^b^	13.5	15.9	14.6	16.4	1.4 (0.93–2.09)	0.217		1.01 (1.00-1.03)	0.090
Males^b^	10.6	9.1	8.9	9.3	1.0 (0.63–1.67)	0.716		1.00 (0.98–1.02)	0.828
**Hyperactivity symptoms score ≥ cut-off**									
Females^b^	16.8	17.9	15.9	18.0	1.2 (0.81–1.72)	0.480		1.00 (0.99–1.02)	0.619
Males^b^	15.0	13.6	13.2	12.5	0.8 (0.54–1.29)	0.746		0.99 (0.98–1.01)	0.278
**Emotional symptoms score ≥ cut-off**									
Females^b^	17.5	21.3	26.3	30.1	**2.1 (1.51–3.05)**	**< 0.001**		**1.04 (1.03–1.05)**	**< 0.001**
Males^b^	6.3	7.0	6.2	6.7	1.3 (0.73–2.30)	0.679		1.01 (0.99–1.03)	0.448
**Conduct problems score ≥ cut-off**									
Females^b^	11.3	10.8	7.5	7.5	0.7 (0.44–1.22)	0.061		0.98 (0.96-1.00)	**0.025**
Males^b^	14.0	14.8	12.6	11.9	0.9 (0.61–1.46)	0.655		1.00 (0.98–1.01)	0.569
**Peer problems score ≥ cut-off**									
Females^b^	20.2	18.1	18.5	21.5	1.1 (0.73–1.51)	0.508		1.00 (0.99–1.01)	0.973
Males^b^	23.4	20.4	17.6	20.0	0.98 (0.69–1.39)	**0.027**		0.99 (0.98-1.00)	0.116
**Prosocial behavior score ≤ cut-off** ^e^									
Females	3.5	8.9	4.9	5.2	1.7 (0.87–3.44)	**< 0.001**		1.01 (0.99–1.04)	0.212
Males	17.5	17.4	13.6	14.0	0.8 (0.54–1.23)	0.050		0.99 (0.97-1.00)	**0.043**
**Alcohol use**									
Females						**< 0.001**			**< 0.001**
≥ Once a month	51.3	31.3	24.4	20.6	**0.2 (0.12–0.24)**			**0.91 (0.90–0.92)**	
≥ Once a week	7.7	5.1	1.6	0.7	**0.1 (0.01–0.15)**			**0.87 (0.85–0.90)**	
Males						< 0.001			< 0.001
≥ Once a month	42.8	32.4	23.6	21.1	**0.3 (0.20–0.41)**			**0.93 (0.92–0.95)**	
≥ Once a week	11.2	6.7	2.6	2.6	**0.1 (0.06–0.28)**			**0.90 (0.88–0.92)**	
**Drunkenness**									
Females						**< 0.001**			**< 0.001**
≥ Once a month	43.7	26.6	18.6	14.5	**0.1 (0.10–0.21)**			**0.91 (0.89–0.92)**	
≥ Once a week	6.1	3.8	0.8	0.6	**0.0 (0.01–0.20)**			**0.87 (0.84–0.90)**	
Males						**< 0.001**			**< 0.001**
≥ Once a month	37.3	24.8	17.2	14.9	**0.2 (0.15–0.33)**			**0.92 (0.91–0.94)**	
≥ Once a week	8.0	4.9	1.7	1.8	**0.1 (0.06–0.34)**			**0.90 (0.88–0.92)**	
**Tobacco use**									
Females						**< 0.001**			**< 0.001**
Seldom	20.8	13.1	11.0	9.5	**0.3 (0.19–0.45)**			**0.93 (0.92–0.95)**	
≥ Once a week	29.2	17.2	8.8	6.5	**0.1 (0.06–0.18)**			**0.90 (0.88–0.91)**	
Males						**< 0.001**			**0.005**
Seldom	12.7	13.5	12.8	9.0	**0.5 (0.30–0.83)**			**0.98 (0.96–0.99)**	
≥ Once a week	25.4	20.4	16.7	12.8	**0.4 (0.29–0.67)**			**0.96 (0.95–0.98)**	

^a^Analyses were adjusted with school grade, family structure and city

^b^≥ cut-off refers to adolescents scoring over about the 90th percentile defined for the whole sample in 1998

^c^Strengths and Difficulties Questionnaire

^d^Bonferroni correction

^e^ ≤ refers to 10th percentile cut-off point

### Changes in substance use

All the results were adjusted for school grade, family structure and city. When comparing the years 1998 and 2018, significant decreases were found in alcohol use, drunkenness and tobacco use among both females and males (Table 2).Percentages of females using alcohol monthly decreased from 51.3 to 20.6% (OR 0.2, 98.75% CI 0.12–0.24) and among males from 42.8 to 21.1% (OR 0.3, 98.75% CI 0.20–0.41). Similarly, being drunk at least once a month decreased among females from 43.7 to 14.5% (OR 0.1, 98.75% CI 0.10–0.21) and among males from 37.3 to 14.9% (OR 0.2, 98.75% CI 0.15–0.33). Smoking tobacco frequently, at least once a week, decreased among females from 29.2 to 6.5% (OR 0.1, 98.75% CI 0.06–0.18) and among males from 25.4 to 12.8% (OR 0.4, 98.75% CI 0.29–0.67). The changes were similar when assessing linear change during the same time period.

## Discussion

Emotional symptoms among females significantly and continuously increased from 1998 to 2018. This trend was not seen among males; their situation was rather stable. There was improvement in prosocial behavior and in peer problems among males. The changes were small, although statistically significant. For example, the number of males in the clinical range of peer problems decreased 3.4%. Another positive finding was that substance use in terms of alcohol use, drunkenness and smoking decreased among males and females over the study period. These trends of emotional problems and substance use were congruent when comparing years and when assessing linear change.

During the 20-year study period there was a consistent increase in the prevalence of females with increased levels of emotional symptoms. Among males, the respective trend has been rather stable. The results are in line with the nationwide SHP study reporting significant increases in anxiety and depressive symptoms among females in Finland during the last ten years [17]. In addition, the worrisome situation especially concerning mental health among females has been observed in other Nordic countries [16].

Increases in internalizing symptoms have been reported in earlier studies [28], especially in females [29], but results on gender differences are mixed [12]. Increases among females may be due to several reasons. Academic pressure and school-related stress has increased, and those typically affect females differently than males. Females tend to feel the effects of school burnout differently than males; particularly, they experience feelings of inadequacy more than males since females may internalize stress [30]. Puberty occurring earlier than before among females [31] is also one factor that may increase or cause earlier occurrence of emotional symptoms [15]. Awareness of mental health has increased, which is a positive change, but can be paradoxically related to this trend, as mental health problems and awareness may affect each other in a cyclical, intensifying manner [32]. In practice, this means that people may interpret and report milder forms of distress as mental health problems, either because of improved recognition or overinterpretation [32]. While emotional symptoms have been on the rise, the amount of time spent online has increased substantially [33], and social media is known to be associated with internalizing symptoms especially among young females [34]. It has been argued that the assumed dimensions of the SDQ do not fully correspond to the mental health problems of mid-adolescents [35]. However, the emotional subscale is found to be functional in identifying depressive or anxiety symptoms among adolescents [36], which further supports the validity of our study results.

We found rather steady situation in mental health problems among adolescents during the twenty-year study period. Interestingly, at the same time there were increases in mental health service use [4] and increased numbers of psychiatric diagnoses in Finland [37]. One positive finding of our study was that prosocial behavior, including helping, sharing and caring for others, improved over time. Improvements were seen among males, and gender difference thus slightly decreased. However, females still reported better prosocial behavior at every time point, compared to males. Another positive finding was that peer problems improved among males. The results are positive considering how prosocial behavior is consistently linked with psychosocial well-being [38] and is essential to positive youth development [39]. Several possibilities may explain the improvements, including increased awareness of the importance of positive parenting [40], changes in youth culture and school environment—since feeling safe at school is related to mental health difficulties [41]—and possibilities for activities outside of school [42]. Another explanation could be that increases in media exposure and online social interactions can foster prosocial tendencies among adolescents [43]. However, social media use can also have a deleterious influence, for example in the form of online aggression or cyberbullying [44].

Our study found a stable trend in hyperactivity among adolescents, notwithstanding that the prevalence of ADHD medication use among children and adolescents has increased significantly in Finland [45, 46] and in the Nordic countries [47]. In Finland, the use of ADHD medication before the year 2000 was almost non-existent [45]. The use level was 0.9% in 2008 among males and further increased to 4.2% by 2018 [46]. Among females, the prevalence was 0.1% in 2008 and 1.3% in 2018 [46]. The present study suggests that medication use is not associated with changes in perceived ADHD symptoms reported by adolescents at the population level.

There was a continuous decrease in alcohol and tobacco use over the whole study period, from 1998 to 2018. These results are in line with those from several other European countries, especially other Nordic countries. For example, the ESPAD study [48] reported a slow but steady decrease in alcohol use among European adolescents. The situation regarding heavy episodic drinking has remained rather stable since 2015 in most Nordic countries, and has even continued decreasing in Norway [48]. An increasing number of adolescents are choosing to completely abstain from drinking alcohol. Those that abstain have better physical and mental health and they perform better in school than their drinking peers [49], making this behavior worth encouraging. Decreases were also seen in cigarette smoking throughout our study period, similar to those at the European level [48]. However, since e-cigarettes rapidly became popular, the overall prevalence of inhaled nicotine use has been rather stable, or even increased [48]. Thus, the trend of substance use continues to be highly important to follow.

## Limitations

Some limitations should be considered when interpreting the results of this study. The data was based on self-reports, and the findings would have been more robust if other informants, such as teachers, would have been included. Multiple informants commonly provide discrepant estimates of an adolescent’s situation, and these discrepant estimates often reflect clinically relevant variations in the situation [50]. The response rates remained at almost the same level each year, with approximately 10–20% of possible respondents not taking part in the study. Many of those who did not respond were absent from school on the day of the survey. It is possible that the data do not reflect adolescents with more severe problems, as school absenteeism is associated with increased mental health problems [51]. However, since the study was conducted using similar methods each time, it is likely that absenteeism occurred similarly at each assessment point, although the absents can differ from the rest of the population. We used 90th percentile cut-off point based on the 1998 sample, as it formed the baseline of the study and we wanted to compare later years to this first assessment point. The last assessment was in 2018. Since then, several societal crises have occurred, and this study does not cover that period.

Although the data was not representative nationwide, it was collected from two medium-sized Finnish cities that reflected the composition of typical Finnish cities. The cities were located in the north and in the south of Finland and comprised both urban and rural areas. The demographical characteristics of the participants, such as sex distribution, ethnic background and family composition, were comparable to the national statistics for that age group in Finland. Obviously, the results cannot be generalized to other countries, and there is a need for cross-cultural time-trend studies to address how different social policies, environmental changes and investments in child and adolescent mental health are associated with changes in psychosocial well-being.

## Conclusions

During the 20-year study period, there was a concerning linear increase in emotional symptoms among females. This finding is important since depression and anxiety are the leading causes of disability among adolescents, and these problems can extend into adulthood, impairing both physical and mental health and limiting their opportunities to live fulfilling lives as adults. Another main finding was the improvement in prosocial behavior and in peer relationships among males, all of which can be associated with enhanced well-being in adolescents. In addition, although the use of alcohol and cigarettes decreased, the possible increased in use of other substances needs to be studied. Public health efforts should remain focused on developing and evaluating strategies for screening and providing low-threshold services for adolescents with mental health symptoms.

## Electronic supplementary material

Below is the link to the electronic supplementary material.

Supplementary Material 1

Supplementary Material 2

Supplementary Material 3

Supplementary Material 4

Supplementary Material 5

Supplementary Material 6

## Data Availability

The data that support the findings of this study are available from the last author, [AS], upon reasonable request.
