# Supplementary material for: Twenty-year changes of adolescent mental health and substance use: a Finnish population-based time-trend study
Source: Eur Child Adolesc Psychiatry. 2024 Jul 10;34(2):685–94. doi: 10.1007/s00787-024-02512-9 (PMC11868224; doi:10.1007/s00787-024-02512-9)
Supplement: Supplementary file 1 — Supplementary Material 1 [file 787_2024_2512_MOESM1_ESM.docx]

**Supplement 1.** Population structure in all of Finland, Rovaniemi, and Salo in 1998 and 2018 based on Statistics Finland’s statistical databases

| **Indicator** | **2018** |
| --- | --- |
| Population  Whole country  Rovaniemi  Salo | 5,517,919  62,922  52,321 |
| Mean age (years)  Whole country  Rovaniemi  Salo | 42.9  41.1  46.1 |
| Gender distribution, males (%)  Whole country  Rovaniemi  Salo | 2,723,290 (49.4%)  30,491 (48.5%)  25,777 (49.3%) |
| Unemployment rate (%)  Whole country  Rovaniemi  Salo | 9.8%  11.1%  12.3% |
| Population with higher education^a^ (%)  Whole country  Rovaniemi  Salo | 1,473,008 (26.7%)  17,563 (27.9%)  11,548 (22.1%) |
| Net wealth of households  Whole country  Northern Finland^b,c^  Western Finland^c^ | 214,802  157,499  199,158 |
| Persons with foreign background  Whole country  Rovaniemi  Salo | 402,619 (7.3%)  2,259 (3.6%)  3,280 (6.3%) |
| Persons with the status of a child:  family with two parents and children  Whole country  Rovaniemi  Salo | 1,000,339  11,111  9,187 |

^a^Defined as post-upper secondary education to be given at higher education institutions or at institutes. Tertiary education includes lowest tertiary education, lower tertiary education, higher tertiary education and doctorate or equivalent education.

^b^Also includes information from Eastern Finland

^c^Also includes information from other cities in the major region
