# Supplementary material for: Twenty-year changes of adolescent mental health and substance use: a Finnish population-based time-trend study
Source: Eur Child Adolesc Psychiatry. 2024 Jul 10;34(2):685–94. doi: 10.1007/s00787-024-02512-9 (PMC11868224; doi:10.1007/s00787-024-02512-9)
Supplement: Supplementary file 2 — Supplementary Material 2 [file 787_2024_2512_MOESM2_ESM.docx]

**Supplement 2.** Pearson correlation coefficients and Cronbach’s alpha in the sub-scales of Strengths and Difficulties Questionnaire in females and males

|  | **Females** | | | | |  | **Males** | | | | |
| --- | --- | --- | --- | --- | --- | --- | --- | --- | --- | --- | --- |
|  | **Emotional**  **symptoms** | **Conduct**  **problems** | **Hyperactivity** | **Peer problems** | **Prosocial behavior** |  | **Emotional**  **symptoms** | **Conduct**  **problems** | **Hyperactivity** | **Peer problems** | **Prosocial behavior** |
| **Emotional**  **symptoms** | 1.00 |  |  |  |  |  | 1.00 |  |  |  |  |
| **Conduct**  **problems** | 0.27 | 1.00 |  |  |  |  | 0.31 | 1.00 |  |  |  |
| **Hyperactivity** | 0.31 | 0.52 | 1.00 |  |  |  | 0.31 | 0.49 | 1.00 |  |  |
| **Peer problems** | 0.37 | 0.15 | 0.10 | 1.00 |  |  | 0.43 | 0.20 | 0.14 | 1.00 |  |
| **Prosocial behavior** | -0.08 | -0.34 | -0.29 | -0.14 | 1.00 |  | -0.04 | -0.34 | -0.28 | -0.15 | 1.00 |
| **Cronbach’s alpha** | 0.70 | 0.55 | 0.69 | 0.58 | 0.63 |  | 0.65 | 0.55 | 0.64 | 0.58 | 0.67 |
