# Supplementary material for: Twenty-year changes of adolescent mental health and substance use: a Finnish population-based time-trend study
Source: Eur Child Adolesc Psychiatry. 2024 Jul 10;34(2):685–94. doi: 10.1007/s00787-024-02512-9 (PMC11868224; doi:10.1007/s00787-024-02512-9)
Supplement: Supplementary file 3 — Supplementary Material 3 [file 787_2024_2512_MOESM3_ESM.docx]

Supplement 3. Regression equations for outcome $Y$ for participant from school $z$ without adjusting covariates.

| Mixed effects binary logistic model with year as categorical variable Outcomes: SDQ scales as binary variables | $g\left( P\left( Y\geq\text{cutpoint} \right) \right)=\beta_{0}+\beta_{1}*x_{2008}+\beta_{2}*x_{2014}+\beta_{3}*x_{2018}+\gamma_{z}$**^a,b,c,d^** |
| --- | --- |
| Mixed effects binary logistic model with year as continuous variable Outcomes: SDQ scales as binary variables | $g\left( P\left( Y\geq\text{cutpoint} \right) \right)=\beta_{0}+\beta_{1}*\text{year}+\gamma_{z}$**^a,b,d^** |
| Mixed effects linear model with year as categorical variable Outcomes: SDQ scales as continuous variables | $Y=\beta_{0}+\beta_{1}*x_{2008}+\beta_{2}*x_{2014}+\beta_{3}*x_{2018}+\gamma_{z}$**^c,d^** |
| Mixed effects multinomial logistic model with year as categorical variable Outcomes: Substance use as three-level variables | $\text{ln}\left( \frac{P(Y=k)}{P(Y=\text{"Not at all"})} \right)=\beta_{0k}+\beta_{1k}*x_{2008}+\beta_{2k}*x_{2014}+\beta_{3k}*x_{2018}+\gamma_{zk}$**^c,d,e,f^** |

**^a^** $g\left( p \right)=\text{ln}(\frac{p}{1-p})$
**^b^** for prosocial score, probability modeled was $P\left( Y\leq\text{cutpoint} \right)$
**^c^** variables $x_{\text{year}}$ are dummy variables ($x_{2008}=1$, if the sample year is 2008 and 0 otherwise)
**^d^** school-wise random intercepts $\gamma_{z}$ are normally distributed
**^e^** $k$ is any outcome option other than the reference level (e.g. “more than once a week” or “more than once a month” for alcohol use)
**^f^** fixed effect coefficients $\beta_{jk}$ and random intercepts $\gamma_{zk}$ estimated separately for outcome options $k$
