# Supplementary material for: Twenty-year changes of adolescent mental health and substance use: a Finnish population-based time-trend study
Source: Eur Child Adolesc Psychiatry. 2024 Jul 10;34(2):685–94. doi: 10.1007/s00787-024-02512-9 (PMC11868224; doi:10.1007/s00787-024-02512-9)
Supplement: Supplementary file 4 — Supplementary Material 4 [file 787_2024_2512_MOESM4_ESM.docx]

**Supplement 5.** Comparison of adjusted dichotomized self-reported problems based on 90^th^ percentile cut-off points of SDQ and substance use in 1998, 2008, 2014 and 2018

|  | **1998** | **2008** | **2014** | **2018** | **2008 vs. 1998** | **2014 vs. 2008** | **2018 vs. 2014** | **2018 vs. 1998** | **Overall**  **year**  ***p*-value^a^** |
| --- | --- | --- | --- | --- | --- | --- | --- | --- | --- |
|  | **%** | **%** | **%** | **%** | **OR (98.75%CI)^a,d^** | **OR (98.75%CI)^a,d^** | **OR (98.75%CI)^a,d^** | **OR (98.75%CI)^a,d^** |  |
| **SDQ^c^ total score** **≥ cut-off** | | | | | | | |  |  |
| Females^b^ | 13.5 | 15.9 | 14.6 | 16.4 | 1.2 (0.83–1.69) | 0.9 (0.68–1.31) | 1.3 (0.85–1.83) | 1.4 (0.93–2.09) | 0.217 |
| Males^b^ | 10.6 | 9.1 | 8.9 | 9.3 | 0.9 (0.57–1.32) | 1.0 (0.68–1.53) | 1.2 (0.72–1.87) | 1.0 (0.63–1.67) | 0.716 |
| **Hyperactivity symptoms** **score ≥ cut-off** | | | | | | | |  |  |
| Females^b^ | 16.8 | 17.9 | 15.9 | 18.0 | 1.0 (0.75–1.44) | 0.9 (0.66–1.24) | 1.3 (0.87–1.81) | 1.2 (0.81–1.72) | 0.480 |
| Males^b^ | 15.0 | 13.6 | 13.2 | 12.5 | 0.9 (0.63–1.29) | 1.0 (0.70–1.38) | 0.9 (0.62–1.44) | 0.8 (0.54–1.29) | 0.746 |
| **Emotional symptoms** **score ≥ cut-off** | | | | | | | |  |  |
| Females^b^ | 17.5 | 21.3 | 26.3 | 30.1 | 1.3 (0.94–1.78) | **1.4 (1.02–1.77)** | 1.2 (0.90–1.67) | **2.1 (1.51–3.02)** | **< 0.001** |
| Males^b^ | 6.3 | 7.0 | 6.2 | 6.7 | 1.1 (0.69–1.87) | 0.9 (0.57–1.45) | 1.3 (0.72–2.16) | 1.3 (0.73–2.30) | 0.679 |
| **Conduct problems** **score ≥ cut-off** | | | | | | | |  |  |
| Females^b^ | 11.3 | 10.8 | 7.5 | 7.5 | 1.0 (0.66–1.48) | 0.7 (0.45–1.04) | 1.1 (0.64–1.82) | 0.7 (0.44–1.22) | 0.061 |
| Males^b^ | 14.0 | 14.8 | 12.6 | 11.9 | 1.1 (0.76–1.56) | 0.9 (0.61–1.19) | 1.0 (0.67–1.55) | 0.9 (0.61–1.46) | 0.655 |
| **Peer problems** **score ≥ cut-off** | | | | | | | |  |  |
| Females^b^ | 20.2 | 18.1 | 18.5 | 21.5 | 0.9 (0.64–1.21) | 1.0 (0.76–1.39) | 1.2 (0.82–1.66) | 1.1 (0.73–1.51) | 0.508 |
| Males^b^ | 23.4 | 20.4 | 17.6 | 20.0 | 0.8 (0.63–1.14) | 0.8 (0.62–1.12) | 1.4 (0.97–1.97) | 1.0 (0.69–1.39) | **0.027** |
| **Prosocial behavior** **score ≤ cut-off**^e^ | | | | | | | |  |  |
| Females | 3.5 | 8.9 | 4.9 | 5.2 | **2.7 (1.51–4.83)** | **0.5 (0.33–0.86)** | 1.2 (0.66–2.20) | 1.7 (0.87–3.44) | **< 0.001** |
| Males | 17.5 | 17.4 | 13.6 | 14.0 | 1.0 (0.75–1.45) | 0.7 (0.52–1.00) | 1.1 (0.72–1.63) | 0.8 (0.54–1.23) | **0.050** |
| **Alcohol use** | | | | | | | |  |  |
| Females |  |  |  |  |  |  |  |  | **< 0.001** |
| ≥ Once a month | 51.3 | 31.3 | 24.4 | 20.6 | **0.3 (0.25–0.45)** | **0.6 (0.48–0.84)** | 0.8 (0.54–1.09) | **0.2 (0.12–0.24)** |  |
| ≥ Once a week | 7.7 | 5.1 | 1.6 | 0.7 | **0.4 (0.20–0.59)** | **0.3 (0.12–0.57)** | 0.5 (0.13–1.87) | **0.1 (0.01–0.15)** |  |
| Males |  |  |  |  |  |  |  |  | **< 0.001** |
| ≥ Once a month | 42.8 | 32.4 | 23.6 | 21.1 | **0.6 (0.43–0.74)** | **0.5 (0.41–0.71)** | 0.9 (0.67–1.34) | **0.3 (0.20–0.41)** |  |
| ≥ Once a week | 11.2 | 6.7 | 2.6 | 2.6 | **0.5 (0.31–0.80)** | **0.3 (0.15–0.53)** | 0.9 (0.38–2.18) | **0.1 (0.06–0.28)** |  |
| **Drunkenness** | | | | | | | |  |  |
| Females |  |  |  |  |  |  |  |  | **< 0.001** |
| ≥ Once a month | 43.7 | 26.6 | 18.6 | 14.5 | **0.4 (0.27–0.48)** | **0.6 (0.42–0.77)** | 0.7 (0.47–1.04) | **0.1 (0.10–0.21)** |  |
| ≥ Once a week | 6.1 | 3.8 | 0.8 | 0.6 | **0.4 (0.20–0.69)** | **0.2 (0.06–0.51)** | 0.7 (0.11–3.72) | **0.0 (0.01–0.20)** |  |
| Males |  |  |  |  |  |  |  |  | **< 0.001** |
| ≥ Once a month | 37.3 | 24.8 | 17.2 | 14.9 | **0.5 (0.36–0.65)** | **0.6 (0.40–0.74)** | 0.9 (0.57–1.27) | **0.2 (0.15–0.33)** |  |
| ≥ Once a week | 8.0 | 4.9 | 1.7 | 1.8 | **0.5 (0.28–0.82)** | **0.3 (0.13–0.59)** | 1.1 (0.39–2.95) | **0.1 (0.06–0.34)** |  |
| **Tobacco use** | | | | | | | |  |  |
| Females |  |  |  |  |  |  |  |  | **< 0.001** |
| Seldom | 20.8 | 13.1 | 11.0 | 9.5 | **0.4 (0.30–0.61)** | 0.7 (0.51–1.05) | 0.9 (0.59–1.45) | **0.3 (0.19–0.45)** |  |
| ≥ Once a week | 29.2 | 17.2 | 8.8 | 6.5 | **0.4 (0.27–0.51)** | **0.5 (0.31–0.66)** | 0.7 (0.37–1.13) | **0.1 (0.06–0.18)** |  |
| Males |  |  |  |  |  |  |  |  | **< 0.001** |
| Seldom | 12.7 | 13.5 | 12.8 | 9.0 | 1.0 (0.67–1.42) | 0.9 (0.62–1.25) | **0.6 (0.35–0.95)** | **0.5 (0.30–0.83)** |  |
| ≥ Once a week | 25.4 | 20.4 | 16.7 | 12.8 | 0.8 (0.56–1.06) | 0.8 (0.56–1.05) | 0.8 (0.49–1.13) | **0.4 (0.29–0.67)** |  |

^a^Analyses were adjusted with school grade, family structure and city

^b^≥ cut-off refers to adolescents scoring over about the 90^th^ percentile defined for the whole sample in 1998

^c^Strengths and Difficulties Questionnaire

^d^Bonferroni correction

^e^ ≤ refers to 10^th^ percentile cut-off point
