# Supplementary material for: Twenty-year changes of adolescent mental health and substance use: a Finnish population-based time-trend study
Source: Eur Child Adolesc Psychiatry. 2024 Jul 10;34(2):685–94. doi: 10.1007/s00787-024-02512-9 (PMC11868224; doi:10.1007/s00787-024-02512-9)
Supplement: Supplementary file 5 — Supplementary Material 5 [file 787_2024_2512_MOESM5_ESM.docx]

**Supplement 6.** Strength and Difficulties Questionnaire subscales 90^th^ percentile cut-off points in 1998, 2008, 2014 and 2018

|  | **1998** | | | | **2008** | | | | **2014** | | | | **2018** | | | |
| --- | --- | --- | --- | --- | --- | --- | --- | --- | --- | --- | --- | --- | --- | --- | --- | --- |
|  | **10^th^** | **90^th^** | **min-max** | **MD** | **10^th^** | **90^th^** | **min-max** | **MD** | **10^th^** | **90^th^** | **min-max** | **MD** | **10^th^** | **90^th^** | **min-max** | **MD** |
| **Total difficulties** | 5 | 18 | 0-38 | 11 | 5 | 18 | 0-39 | 1 | 5 | 18 | 0-31 | 10 | 5 | 19 | 0-34 | 10 |
| **Hyperactivity** | 1 | 6 | 0-10 | 4 | 1 | 6 | 0-10 | 3 | 1 | 6 | 0-10 | 3 | 1 | 6 | 0-10 | 3 |
| **Emotional symptoms** | 0 | 6 | 0-10 | 3 | 0 | 6 | 0-10 | 3 | 0 | 6 | 0-10 | 3 | 0 | 7 | 0-10 | 3 |
| **Conduct problems** | 1 | 5 | 0-10 | 2 | 1 | 5 | 0-10 | 2 | 1 | 5 | 0-9 | 2 | 0 | 4 | 0-10 | 2 |
| **Peer problems** | 1 | 4 | 0-10 | 2 | 0 | 4 | 0-10 | 2 | 0 | 4 | 0-10 | 2 | 0 | 5 | 0-10 | 2 |
| **Prosocial behavior** | 4 | 9 | 0-10 | 7 | 4 | 9 | 0-10 | 7 | 5 | 9 | 0-10 | 7 | 5 | 9 | 0-10 | 7 |
