# Supplementary material for: Twenty-year changes of adolescent mental health and substance use: a Finnish population-based time-trend study
Source: Eur Child Adolesc Psychiatry. 2024 Jul 10;34(2):685–94. doi: 10.1007/s00787-024-02512-9 (PMC11868224; doi:10.1007/s00787-024-02512-9)
Supplement: Supplementary file 6 — Supplementary Material 6 [file 787_2024_2512_MOESM6_ESM.docx]

**Supplement 4.** Comparison of self-reported problems based on the Strengths and Difficulties Questionnaire in 1998, 2008, 2014 and 2018

|  | **Females** | | | | **p value of pairwise year comparisons^a,c^** | | | | **Males** | | | | **p value of pairwise year comparisons^a,c^** | | | |
| --- | --- | --- | --- | --- | --- | --- | --- | --- | --- | --- | --- | --- | --- | --- | --- | --- |
|  | **1998**  **Mean (SD)** | **2008**  **Mean (SD)** | **2014**  **Mean (SD)** | **2018**  **Mean (SD)** | **2008 vs. 1998** | **2014 vs. 2008** | **2018 vs. 2014** | **2018 vs. 1998** | **1998**  **Mean (SD)** | **2008**  **Mean (SD)** | **2014**  **Mean (SD)** | **2018**  **Mean (SD)** | **2008 vs. 1998** | **2014 vs. 2008** | **2018 vs. 2014** | **2018 vs. 1998** |
| **SDQ^b^ Total** | 11.7  (5.2) | 11.6  (5.5) | 11.7  (5.4) | 12.0  (5.4) | 1.000 | 1.000 | 0.321 | 0.338 | 10.8  (5.3) | 10.3  (5.2) | 9.9  (4.9) | 9.8  (5.2) | 0.219 | 1.000 | 1.000 | 0.248 |
| **Hyperactivity** | 3.7  (2.1) | 3.4  (2.2) | 3.5  (2.1) | 3.4  (2.2) | **0.016** | 1.000 | 1.000 | 0.461 | 3.5  (2.0) | 3.3  (2.1) | 3.3  (2.0) | 3.3  (2.0) | 0.182 | 1.000 | 1.000 | 1.000 |
| **Emotional**  **symptoms** | 3.5  (2.2) | 3.7  2.3 | 4.0  2.4 | 4.3  2.4 | **0.127** | **0.045** | **0.017** | **<0.001** | 2.3  (1.9) | 2.2  (1.9) | 2.2  (1.9) | 2.1  (1.9) | 1.000 | 1.000 | 1.000 | 1.000 |
| **Conduct**  **problems** | 2.3  (1.7) | 2.4  (1.7) | 2.1  (1.6) | 2.0  (1.6) | 0.542 | **0.003** | 1.000 | 0.065 | 2.5  (1.8) | 2.6  (1.8) | 2.5  (1.6) | 2.4  (1.7) | 1.000 | 0.985 | 1.000 | 1.000 |
| **Peer problems** | 2.3  (1.6) | 2.0  (1.8) | 2.1  (1.7) | 2.3  (1.8) | **0.095** | 1.000 | 0.361 | 1.000 | 2.5  (1.7) | 2.2  (1.8) | 2.0  (1.7) | 2.0  (1.8) | **0.002** | 0.503 | 1.000 | **0.002** |
| **Prosocial behavior** | 7.3  (1.6) | 7.1  (1.8) | 7.5  (1.8) | 7.6  (1.8) | 0.089 | **<0.001** | 1.000 | 0.109 | 6.0  (1.9) | 6.1  (1.9) | 6.5  (1.9) | 6.6  (2.0) | 1.000 | **<0.001** | 1.000 | **<0.001** |

**^a^** Adjusted for school grade, family structure and city

**^b^** Strengths and Difficulties Questionnaire

^c^ p-values multiplied by 4 to adjust for multiple tests
